# A teacher's lens on student motivation: a three-year longitudinal study of NCS students' Chinese writing motivation in Hong Kong

**DOI:** 10.3389/fpsyg.2026.1906908

**Published:** 2026-08-11

**Authors:** Shanshan Chen, Jiangshan Sun, Wenyue Hu, Juan Zou, Tiantian Jin

**Affiliations:** 1 School of Information Technology in Education, South China Normal University, Guangzhou, China; 2 Chinese Academy of Education Big Data, Qufu Normal University, Qufu, Shandong, China; 3 Department of Early Childhood Education, Faculty of Education and Human Development, The Education University of Hong Kong, Hong Kong, Hong Kong SAR, China; 4 Department of Psychology, South China Normal University, Guangzhou, China

**Keywords:** Chinese as a second language, NCS students, self-determination theory, student motivation, teacher-researcher

## Abstract

Motivation is fundamental to foreign language learning, yet little longitudinal evidence exists on how young multilingual learners' writing motivation evolves over time, particularly in the Asia-Pacific region. To address this gap, this 3-year teacher-researcher study tracked 25 non-Chinese speaking (NCS) primary students' Chinese writing motivation from Grade 4 to Grade 6, guided by Self-Determination Theory. The study addressed two questions: (1) How do motivational orientations evolve across technology-enhanced and experiential writing tasks? (2) What classroom conditions support or undermine competence, autonomy, and relatedness? Employing a longitudinal teacher-researcher methodology, the study included 38 reflective teaching notes, 100 student writing samples across four task conditions (the Prezi virtual tour administered in Grade 4; regular curriculum narrative writing in Grade 5; and VR immersion, a physical field trip, and an independent AI-image task administered in Grade 6), and student questionnaires. The analysis revealed three developmental paths. Competence grew as writing shifted from basic visual descriptions to rich multisensory expression. Autonomy manifested when students integrated personal and cultural connexions, enabled by teacher affirmation. Relatedness anchored motivation during collaborative physical visits. However, the final independent AI-image task revealed stark individual differences: only students with sustained need satisfaction maintained rich writing; others regressed, showing that prior positive experiences alone do not guarantee internalised motivation.

## 1 Introduction

Maintaining writing motivation of learners with varying language backgrounds is a longstanding concern in education worldwide. In Hong Kong, this challenge is particularly evident among non-Chinese-speaking (NCS) students, who must acquire Chinese reading and writing skills in a multilingual teaching environment (Loh and Tam, 2022). Although digital tools are widely regarded as a panacea for stimulating learning motivation (Makransky and Petersen, 2021), there is still an empirical debate over whether they can cultivate intrinsic motivation - the sustained driving force for writing development. What is crucial is that we lack a detailed longitudinal understanding of how and why the motivation orientation of young learners evolves with a series of technological experiences. This gap has serious consequences: without sustained writing motivation, NCS students are at risk of falling behind in their Chinese literacy development, limiting their academic progress and future opportunities in Hong Kong's multilingual society.

While Self-Determination Theory (SDT; Ryan and Deci, 2000) offers a robust framework for understanding motivation through the satisfaction of competence, autonomy, and relatedness, its application to technology-assisted L2 writing remains limited in several critical respects. Current research predominantly relies on short-term, cross-sectional designs, leaving the dynamic evolution of writing motivation across multiple school years largely unexplored (Dong, 2025). Furthermore, empirical evidence regarding how different technology modalities support or hinder basic psychological needs among young multilingual learners in the Asia-Pacific context remains scarce. Crucially, the literature often overlooks the unique capacity of teacher-researchers to construct and examine the motivational ecology from within the classroom.

In order to address these interwoven gaps, this 3-year longitudinal study tracked 25 NCS students as they progressed from fourth grade to sixth grade. The study examined their motivation for writing in Chinese in a range of carefully designed instructional tasks assisted by different technologies: from multimedia presentations (Prezi) and immersive tools (VR) to generative artificial intelligence applications that offer new possibilities for enriching students' learning experiences. To be specific, the four task conditions were implemented at different points across the t3-year period: the Prezi virtual tour was administered in Grade 4; in Grade 5, students produced narrative writing samples as part of their regular curriculum (no technology-mediated intervention); and the VR immersion, physical field trip, and AI-image writing task were administered in Grade 6. This design enables us to go beyond the level of “technology can stimulate learning motivation,” and further to explore the dynamic development and the internalisation of motivation. This study is based on SDT, and revolves around the following two research questions:

1. How do NCS students' motivational orientations towards Chinese writing evolve across different technology-mediated writing tasks?
2. What classroom conditions and task characteristics support or undermine their basic psychological needs for competence, autonomy, and relatedness?

## 2 Literature review

### 2.1 Self-determination theory and L2 writing motivation

SDT proposed by Deci and Ryan (2000) provides a comprehensive framework for understanding human motivation. This theory identifies three fundamental psychological needs, arguing that fulfilling these needs is necessary for optimal functioning: competence (sense of control and efficacy), autonomy (volitional experience and psychological freedom), and relatedness (a sense of connexion and belonging with others). When these needs are met, individuals are more likely to engage in activities out of intrinsic motivation and maintain sustained interest (Ryan and Deci, 2020). In the field of education, an environment that meets these needs can predict a series of positive outcomes, including engagement, perseverance, and wellbeing (Niemiec and Ryan, 2009).

In recent years, the application of SDT in second language (L2) learning has witnessed a significant increase. Alamer et al. (2025) conducted a landmark meta-analysis that synthesised the results of multiple studies on the application of SDT in the L2 context, revealing a positive correlation between autonomous motivation (including intrinsic motivation and identity regulation) and L2 academic performance (weighted *r* = 0.23), while there was no significant correlation between control motivation (including extrinsic regulation and introjected regulation) and L2 academic performance. Moreover, autonomous motivation was negatively correlated with language anxiety (*r* = −0.23), highlighting the protective role of need satisfaction in L2 learning.

In the field of L2 writing, research based on SDT has explored how teaching conditions can either promote or undermine learning motivation. Teng et al. (2024) conducted a longitudinal study that tracked the motivation trajectories of L2 writers at three time points. The results showed that students who received supportive autonomy feedback demonstrated more sustained learning engagement and more mature writing strategies over time. Their qualitative case studies show how learners internalise feedback differently depending on whether their psychological needs for competence, autonomy, and relatedness are met.

Despite these advancements, significant gaps remain. Most L2 studies based on SDT have focused on the Western context where English is the foreign language. There remains a critical need to examine how psychological needs are met among young multilingual learners acquiring Chinese in the Asia-Pacific region.

### 2.2 Technology-mediated learning and the teacher's role

The application of technology in language education has opened up new avenues for stimulating learners‘ motivation, but it has also brought about complexity regarding how digital tools interact with the psychological needs of learners (Makransky and Petersen, 2021). Using an SDT perspective, emerging research explores how various digital tools, from virtual reality to artificial intelligence, foster or impede the satisfaction of learners' basic psychological needs.

**Virtual Reality (VR)** offers the unique advantage of supporting capability enhancement through an immersive, embodied experience that provides rich sensory input and instant feedback (Makransky and Petersen, 2021). Studies have shown that a VR experience can increase learners' confidence and willingness to engage in challenging content, especially when the technology is well integrated with the teaching scaffolding. A review by (Alé and Arancibia 2025) systematically analysed the evidence of incentive strategies driven by emerging technologies, reporting that the overall effect size of technology-enhanced incentive interventions was **ES** **=** **0.886**. The review compared different types of technologies and found that immersive technologies (VR/XR) had a stronger impact on intrinsic motivation than non-immersive tools, possibly because they can simultaneously meet the needs of competence and autonomy. However, the authors emphasised that technology alone is insufficient—the quality of pedagogical design and teacher-researcher mediation critically determines motivational outcomes.

Under the SDT framework, teachers play a crucial social role, as they can both support students‘ psychological needs and undermine them (Reeve and Cheon, 2021). This role appears particularly critical in technology-assisted settings, where digital tools alone are insufficient to sustain intrinsic motivation without thoughtful pedagogical orchestration. Adopting a teacher-researcher perspective, Nakata et al. (2022) demonstrated how a language teacher's reflective practises and pedagogical adjustments continuously reshape classroom conditions to meet learners' psychological needs. However, how teacher-researchers navigate this technology-mediated motivational ecology over extended periods remains underexplored.

### 2.3 The longitudinal gap

Although recent studies have begun adopting time-series designs to capture dynamic motivational shifts (e.g., Dong, 2025), these interventions typically span only short durations, leaving a significant gap in understanding how writing motivation evolves across multiple school years. To address this gap, the present study tracked 25 NCS students from Grade 4 to Grade 6 across varying technology-assisted tasks (Prezi presentations, virtual reality, field trips, and AI-generated images)—a timeframe that remains rare in L2 motivation research. Acknowledging the Grade 5 interval as a non-intervention observational phase with narrative writing from the regular curriculum, this multi-wave design responds directly to the call for extended longitudinal investigations in authentic L2 classroom settings (Teng et al., 2024).

### 2.4 Investment, identity, and the teacher-researcher role

This study extends Norton's (2013) investment theory by examining the teacher-researcher's role in the motivational ecology. We propose the concept of reciprocal investment as an extension of investment theory, capturing the bidirectional and cyclical dynamics. Darvin and Norton (2023) distinguished between investment and motivation, arguing that motivation is a psychological construct concerned with cognitive and affective factors, whereas investment is primarily sociological, focusing on how histories, lived experiences, and social practises shape language learning. They noted that investment signals how conditions of power impinge on the desire of learners to learn and practise the target language (Darvin and Norton, 2023; p. 30). Importantly, the models proposed by (Darvin 2015; 2023) place investment at the intersection of identity, capital and ideology, acknowledging that learners negotiate their identities in different social spaces, and that their investments are influenced by the extent to which their language and cultural resources are recognised as legitimate by those in power.

In multilingual classrooms, learners bring varying linguistic and cultural resources—such as home languages (e.g., Urdu, Hindi, Nepali), local Hong Kong life experiences, and cross-cultural engagement with Chinese heritage (e.g., visits to historic landmarks like Kowloon Walled City Park)—into their learning, making the recognition of these resources as legitimate capital crucial for learning outcomes (Darvin and Norton, 2019). This recognition does not occur naturally; rather, it requires teaching decisions to affirm the learners' identity and mobilise their existing knowledge, transforming it into the motivation for further learning. However, the existing investment framework mainly focuses on how learners deal with power relations in their interactions with teachers, institutions and the target language community. It pays less attention to how teachers themselves engage in the incentive ecosystem of the classroom, and how this engagement is in turn influenced by student feedback.

This study expands the investment theory by examining the role of teachers as researchers in the incentive ecosystem. We propose the concept of reciprocal investment to capture the bidirectional and cyclical dynamics, where the teaching investment made by teacher-researchers—formed by professional knowledge, background constraints, and continuous observation of student participation—promotes the satisfaction of students' needs and the development of their motivation, thereby fostering teachers' professional commitment and further teaching innovation. This concept is based on the observation made by Norton and (Norton and Toohey 2011, p. 421), namely that language learners may have strong motivations, but if the classroom language practise fails to adequately meet their needs, they may not invest enough in the classroom language practise. We extend this insight to teachers: Teachers also decide whether to invest in or abandon specific teaching methods based on students' reactions and whether these methods can lead to effective participation. Therefore, reciprocal investment provides a theoretical perspective for understanding the co-evolution of teachers' practises and students' motivations in classroom research.

## 3 Methods

### 3.1 Research design and researcher positionality

This study employed a longitudinal teacher-researcher case study design to track the learning progress of the same group of students from fourth grade to sixth grade (the 2022–2025 academic year). The first author held the dual roles of classroom teacher and doctoral researcher. This identity enabled the researcher to conduct continuous, contextualised observations and adjust teaching methods in real time based on emerging insights—we conceptualise this dynamic process as reciprocal investment in the classroom motivation ecosystem (Nakata et al., 2022). To reduce potential biases, the researchers selected a 20% data subset for inter-coder reliability testing (Cohen's κ = 0.8049), and regularly discussed the research results with fellow researchers. All student names were pseudonyms. In order to maintain the rigour of the analysis and eliminate potential biases that may arise from the dual role of teacher and researcher, the first author consistently wrote reflection logs throughout the entire research process. These logs documented teaching decisions, hypotheses regarding students' motivation, and reflections on how the dual role of teacher and researcher affected data collection and interpretation. These reflections were shared with collaborators at regular peer discussion sessions, actively seeking and discussing alternative interpretations of the data. This process helped to distinguish between the teacher's teaching observations and the researcher's analytical conclusions.

### 3.2 Participants and context

The participants were 25 NCS students (12 girls and 13 boys) studying at a government-funded English primary school in Hong Kong. Their native languages included Urdu (*n* = 12), Hindi (*n* = 8), and Nepali (*n* = 5). All participants received 5–6 h of Chinese as a second language instruction per week, while the teaching language for all other subjects in the school was English. The school's students are predominantly from low-income families, with more than 60% receiving government financial aid, making these students educationally disadvantaged and making their learning motivation trajectories important for understanding equity in technology-assisted learning.

This study adopted a convenience sampling strategy, which was in line with the teacher-researcher design. The reason for choosing this school was that the student population there was mainly composed of NCS students, and it was also the workplace of the first author. This enabled the first author to continuously enter the classroom and assume the crucial dual role of teacher-researcher in this study. This convenience sampling approach aligns directly with the qualitative teacher-researcher paradigm, as it provided sustained, authentic access to the natural classroom environment over a 3-year period, prioritising ecological validity and contextual depth over statistical generalisability. At the beginning of the research, all 25 NCS students in the fourth grade of this school were invited to participate, and all of them agreed. Although the sample was not randomly selected, for a longitudinal case study, the sample size was appropriate because the study prioritised analysis depth and ecological validity rather than statistical universality.

It is worth noting that, in line with the common practise in Hong Kong primary schools, the students participating in the study had different Chinese teachers between the fourth and sixth grades. The first author taught the fourth and sixth graders, while another Chinese teacher taught the fifth graders. During this year, the students created narrative writing works (e.g., “A Person I Respect” and “My Mother”) in regular Chinese classes. Although these fifth-grade works were not travelogues, they provided valuable longitudinal evidence for the students' overall development of writing skills during the period of natural teacher changes—a phenomenon that is common in longitudinal studies of L2 learning motivation but rarely studied.

It should also be noted that the availability of data varies at different time points. Although the main focus of the travelogue writing task was in the fourth and sixth grades, the data for the fifth grade included narrative writing samples from regular courses rather than specific technology-assisted writing tasks. This reflects the real teaching environment in Hong Kong primary schools, where the teaching tasks and course priorities of teachers do not remain completely consistent across different grades.

### 3.3 The writing task sequence and data collection

The study comprised three waves of data collection across the 3-year period. In Grade 4, students completed one travelogue writing task supported by a Prezi virtual tour, which served as the baseline. No technology-mediated writing intervention was implemented in Grade 5; students produced narrative writing samples (e.g., ‘A Person I Respect' and ‘My Mother') as part of their regular Chinese curriculum. In Grade 6, three writing tasks were administered: a short VR-supported writing task on the Forbidden City, a full travelogue following a physical field trip to Kowloon Walled City Park, and an independent AI-image writing task, which served as the final summative evaluation of motivational internalisation. This design allows us to trace motivational development across a 3-year period while acknowledging the Grade 5 interval as a non-intervention observational phase. The data is derived from three main sources, covering a series of four Chinese travelogue writing tasks, each of which is assisted by different technologies or teaching methods (see Table 1 for an overview). During and after the field study at the Kowloon City Walled City Park, the students used cameras to record the interesting attractions they observed. After the visit, they selected and printed the photos, and incorporated them into the hand-drawn map of the park. This mixed-media map-making task provided visual and spatial references for their subsequent travelogue writing, ingeniously combining digital records with physical presentation, which not only helps with the recall of memories but also aids in the organisation of narratives.

1. **Student Writing Samples**: A corpus of 100 student essays (4 tasks × 25 students) and 25 mixed-media maps from the field trip were collected, transcribed, and anonymised. These maps were drawn by the students. They combined hand-drawn sketches with the printed photos taken during the visit, and pasted these photos onto the maps as visual reference points for their subsequent writing.
2. **Teacher Reflective Notes**: The first author maintained 38 entries of reflective notes throughout the three years. These documented classroom observations, student comments, pedagogical decisions, and reflections on individual motivational trajectories.
3. **Student Questionnaires**: Two complementary questionnaires were administered at the end of the Grade 4 unit. The Travelogue Unit Evaluation (5-point Likert scale, *n* = 21) measured students' perceived learning gains, tool effectiveness, and skill improvement. Descriptive analysis showed that the physical field trip was rated as highly helpful (M = 4.2381, SD = 1.1360), whereas the Prezi virtual tour received more varied ratings (M = 3.3810, SD = 1.4310). Open-ended responses (e.g., “the sound of water falling,” “the smell of flowers”) were transcribed and thematically analysed. The Inquiry-Based Learning Perception Scale (5-point Likert scale, *n* = 21) captured students' engagement with experiential learning; 42.9% of students (*n* = 21) reported discovering “many” or “very many” new details during the field trip that were absent from the Prezi tour. On the day of the questionnaire survey, 4 students were absent. As a result, 21 valid questionnaires were obtained. All questionnaire data were processed using Microsoft Excel.

**Table 1:** Longitudinal sequence of writing tasks and data collection.

Time point	Task description	Technological/pedagogical support	Data collected
Grade 4 (2022–23)	Travelogue on Hong Kong Park	Prezi virtual tour (visual-dominant)	Student essays (*n* = 25)
Grade 5 (2023–24)	Narrative writing: “A Person I Respect” and “My Mother”	Regular Chinese curriculum	Student essays (*n* = 25)
Grade 6 (2024–25)	Short writing on the Forbidden City	VR immersive experience	Student essays (*n* = 25)
Grade 6 (2024–25)	Full travelogue on Kowloon Walled City Park	Physical field trip + mixed-media maps (hand-drawn sketches with printed photographs)	Student essays (*n* = 25) + Maps (*n* = 25)
Grade 6 (2024–25)	Examination essay on Jinshanhu Park	AI-generated image (no teacher scaffolding)	Student essays (*n* = 25)

Grade 5 writing samples were produced as part of the regular Chinese curriculum under a different Chinese language teacher, reflecting the authentic Hong Kong primary school context where teacher assignment across grades is not necessarily continuous. These narrative writing samples provide longitudinal evidence of students' general writing development during the interval between the Grade 4 and Grade 6 focal travelogue tasks.

### 3.4 Data analysis

Data analysis proceeded in three integrated phases:

1. **Longitudinal Thematic Analysis:** This study employed NVivo 12 software for longitudinal thematic analysis (Thomson and Holland, 2003). The coding followed the three requirements of SDT: competence (such as sensory details, syntactic complexity), autonomy (such as personal expression, cultural connexion), and relevance (such as collective pronouns, interaction descriptions). Two coders independently coded 20% of the data, achieving a high inter-coder reliability (Cohen's κ = 0.8049). In case of disagreement, it was resolved through negotiation and discussion. Table 2 provides an example of the coding scheme. The thematic analysis follows a six-stage process (Braun and Clarke, 2006): familiarising with the data, generating initial codes, identifying themes, reviewing themes, defining and naming themes, and completing the final analysis. The initial themes were derived from the Self-Determination Theory (SDT) framework (competence, autonomy, and relatedness). Through repeated reading of the data, other themes were identified, such as “cultural identity negotiation” and “teacher affirmation.” The two coders met regularly to compare the coding results and refine the theme definitions. Disagreements (less than 5% of the total number of codes) were resolved through discussion until consensus was reached.
2. **Questionnaire Analysis:** Descriptive statistics and thematic analysis of open-ended responses were employed to triangulate and contextualise the qualitative findings in the writing samples and notes. Specifically, questionnaire data was compared with the evidence from the writing samples and teacher notes to identify areas of convergence and divergence. A comparison between the initial Grade 4 Travelogue Unit Survey and the Grade 6 End-of-Unit Survey (Table 3) illustrates these distinct developmental paths. For instance, the scores given by students on the usefulness of Prezi tools were compared with their actual writing performance in the fourth grade, and their self-reported writing challenges were examined based on the obvious difficulties in their essays.
3. **Case Selection:** To illustrate the spectrum of motivation trajectories, we selected two focal students for in-depth longitudinal analysis: Student T, who showed continuous motivational growth, and Student D, who demonstrated limited progress and regression in the final task. In addition, four other students (Student E, Student A, Student S and Student N) are presented in the Findings to further illustrate the range of trajectories observed across the cohort.

**Table 2:** Illustrative coding scheme for SDT needs.

SDT Need	Code	Example from Student Writing (Grade 6)
Competence	Multisensory detail	“我觸碰著柔軟的座位, 聞到香香的樹木氣息” (I touched the soft seat and smelled the fragrant trees)
Autonomy	Cultural/personal connexion	“看著那些白色的小雕像, 我想起了有一次爸爸帶我去印度寺廟” (Looking at those white statues, I remembered the time my father took me to an Indian temple)
Relatedness	Collective pronoun/shared experience	“我們一路上都在討論周圍的景色” (We discussed the surrounding scenery all along the way)

**Table 3:** Longitudinal comparison of survey responses: student t vs. student a (grade 4 travelogue unit survey to grade 6 end-of-unit survey).

Domain	Grade 4 travelogue unit survey		Grade 6 end-of-unit survey	
Focal student	Student T	Student A	Student T	Student A
Interest and attitude	5/5	3/5	5/5	4/5
Cultural connectedness	—	—	5/5	3/5
Writing self-efficacy	5/5	3/5	5/5	4/5
Tool/modality helpfulness	5/5 (Prezi)	3/5 (Prezi)	VR/Field Trip	Travel/experiential
Qualitative focus	Sensory details	Basic landscape	Personal heritage connexion	Factual history
Overall score	90/100	70/100	95/100	75/100

All pseudonyms are anonymised. Student T represents sustained high-internalisation; Student A represents moderate growth but partial internalisation, remaining reliant on factual description.

### 3.5 Ethical considerations

This study has received ethical approval from the relevant institutional review board. The parents of all participants signed written informed consent forms, and the students themselves also expressed their consent. All data have been anonymised and pseudonyms have been used throughout the text. The role of the researcher as a teacher has been transparently explained to all participants.

## 4 Findings

This chapter presents the findings of a qualitative longitudinal study that focused on the three fundamental psychological needs proposed by SDT: competence, autonomy, and relatedness. For each of these needs, we tracked the evolution of students' motivational orientations through four writing tasks from the fourth grade to the sixth grade, and cited evidence such as students' writing samples, teachers' reflection notes, and students' questionnaires. Finally, this chapter analyses the independent AI-image task, which revealed stark individual differences and evaluated students' internalisation of motivation.

Over a period of 3 years, the development of students' motivation exhibited a clear pattern. In the fourth grade, students wrote with the assistance of the Prezi virtual tour, which was characterised by stylised descriptions, prominent visual effects, and a high degree of consistency with the provided content. The students' writing was mainly for the completion of tasks rather than out of genuine engagement. By the sixth grade, after experiencing virtual reality technology and field visits, students' writing showed a richer sensory experience, a more distinct personal style and social awareness. However, the AI-image tasks done independently revealed significant individual differences, suggesting that not all students internalised motivation evenly.

Table 4 presents a quantitative overview of key indicators across the four writing tasks, illustrating the general trajectory of development. Importantly, the quantitative regression in text length (receding to 203 words) and sensory vocabulary (dropping to 4.9 per 100 words) during the final task should not be misconstrued as a pedagogical failure. Rather, this contraction provides vital longitudinal insights into motivational development, as manifested through the task's unique deployment within a high-stakes, timed Chinese language examination. Stripped of all prior experiential scaffolding—such as VR immersion or physical field trips—and deprived of real-time teacher mediation, students confronted this summative assessment under strict time constraints, relying solely on explicit writing prompts and a descriptive reference map. This pressurised testing environment effectively functioned as a rigorous crucible for motivational internalisation. It brought autonomous motivation into sharp relief: while resilient learners independently synchronised their acquired multisensory and cultural schemata under exam pressure, less autonomous peers regressed to formulaic, enumerative prose. This divergence firmly illustrates the value of timed examination essays as an authentic litmus test for true motivational internalisation.

**Table 4:** Overview of writing indicators across tasks.

Indicator	Grade 4 (Prezi)	Grade 6 (VR)	Grade 6 (physical visit)	Grade 6 (AI-image)
Average length (words)	182	215	247	203
Sensory words per 100 words	2.3	5.1	7.8	4.9
First-person pronouns (%)	62%	71%	84%	68%
Collective pronouns (we/our) (%)	8%	15%	32%	12%

Sensory words include visual, auditory, tactile, olfactory, and kinesthetic descriptors. All figures in Table 4 are descriptive summaries only; no inferential statistics were conducted.

### 4.1 Trajectory 1: developing competence through multisensory writing

The developmental trajectory is perhaps best illustrated by examining a single student, Student E, across the three-year period. In Grade 4, writing about Hong Kong Park, she produced a simple, visually-focused description: “我看到了五彩的錦鯉, 水很清澈” (I saw colourful koi fish, the water was very clear). By Grade 6, after the VR immersion and physical field trip to Kowloon Walled City Park, her writing had transformed dramatically, incorporating tactile and olfactory dimensions: “我觸碰著柔軟的座位,聞到香香的樹木氣息,” (I touched the soft seat and smelled the fragrant trees). This shift from purely visual to multisensory narration signifies marked growth in linguistic competence and expressive confidence.

Questionnaire data corroborate this finding. When asked “What details did you discover through the field trip that were not mentioned in the Prezi VR?,” 42.9% of students (*n* = 21) reported discovering “many” or “very many” new details. Their open-ended responses included:

- “The sound of water falling”
- “The smell of flowers”
- “Touching the soft seats”
- “Birds singing”
- “The temperature of the water”

These responses indicate that students themselves recognised the gap between virtual and real experience—and that this recognition may have motivated them to attend more carefully to sensory details in their subsequent writing.

### 4.2 Trajectory 2: asserting autonomy through personal-cultural connexions

Grade 6 writing revealed students' growing autonomy through the proactive integration of personal experiences and cultural backgrounds. Student T, a student of South Asian heritage, wrote about park statues: “看著那些白色的小雕像, 我想起了有一次爸爸帶我去印度寺廟” (*Looking at those white statues*, ***I remembered*** *the time my father took me to an Indian temple*). Teacher-researcher reflective notes documented her initial hesitation:

Student T came to me during the visit and said, “老師,這些雕像讓我想起印度廟裡的神像！"我問她要不要寫進作文裡,她有點猶豫:"可以嗎？這不是中國的東西。"我說當然可以,這是你的真實感受。她笑得很開心。 ” (Teacher-researcher note, October 2024).

This interaction captures the negotiation involved in autonomy development. Student T's hesitation (“可以嗎？這不是中國的東西”) and her teacher-researcher's affirmation (“當然可以”) created space for her to claim authorship over her own experience—a shift from writing-as-compliance to writing-as-meaning-making.

The Grade 4 questionnaire data provide insight into students' baseline sense of autonomy. When asked about their favourite part of the unit, responses were evenly split among teacher-provided options (人工湖, 兒童遊樂場, 觀鳥園, 奧林匹克廣場), suggesting that students had not yet developed strong individual preferences. By contrast, Grade 6 writing revealed more differentiated personal voices.

### 4.3 Trajectory 3: anchoring motivation in social relatedness

The physical field trip notably fostered **relatedness**. Students' writing frequently employed collective pronouns and descriptions of social interaction. For example, 王文 wrote: “我和同學們都感到很神清氣爽” (*My classmates and I all felt refreshed*); Elsha described how “我們一路上都在討論周圍的景色” (*we discussed the surrounding scenery all along the way*). Teacher-researcher notes observed students spontaneously forming small groups, sharing discoveries, and collectively deciding what to include in their writing.

The Grade 4 questionnaire data provide additional evidence of students' valuing of relatedness. When asked what skills they improved through the field trip, 23.8% of students (*n* = 21) selected “合作能力” (collaboration skills)—the third most frequently chosen option after “觀察能力” (observation skills, 33.3%) and “多感官描寫” (multi-sensory description, 28.6%). This indicates that even in Grade 4, students were aware that learning with others was qualitatively different from learning alone.

### 4.4 The crucible: divergent outcomes in the independent AI-image task

The final AI-image writing task, administered without scaffolding, revealed stark individual differences in the internalisation of motivation. Students like Student T, who had experienced sustained need satisfaction across previous tasks, maintained rich writing in the independent context, even employing creative metaphors (e.g., “人工湖像一面鏡子”—*The artificial lake* ***was*** ***like a mirror***). In contrast, students whose needs had been less consistently met reverted to the “thing language” of Grade 4: “看到很多東西,有花,有樹,很開心” (*saw many things, there were flowers, trees, [I] was happy*).

This divergence was not binary. Other students, such as Student A (pseudonym), exemplified the extreme of non-internalisation. His entire post-AI-image essay read: “我們在童樂園欣賞了石雕,然後來到奕園休息” (We admired the stone sculptures at the Children's Playground, then came to Yiyuan to rest). This ‘checklist' approach—listing locations devoid of any sensory or emotional engagement—stands in stark contrast to Student T's metaphorical narrative, revealing a complete absence of internalised motivation when left without pedagogical scaffolding.

To illustrate the various trajectories of motivation development beyond the aforementioned extreme cases, this article briefly describes two additional cases. Student S is a Nepali language student, and he demonstrated stable but moderate progress over a period of three years. In Grade 4, his writing about Hong Kong Park showed basic descriptive competence—he used adjectives such as “清” (clear) and “舒服” (comfortable), and employed causal sentence structures (“因為湖水乾淨,所以看起來很美麗” — because the lake water was clean, it looked very beautiful). The writing followed a clear tour itinerary. By Grade 6, his travelogue on Kowloon Walled City Park incorporated more varied vocabulary (“安靜”, “古色古香”, “翠綠”, “可愛”) and demonstrated awareness of the “step-shift” (spatial progression) structure. However, his description remains largely at the visual level and is more general, lacking the multisensory depth and rich metaphor exhibited in Student T's writing. In the final AI task, his writing, while retaining its basic descriptive power, reverted to a more stylised structure, lacking the creative exposition that characterised students who consistently had their needs met.

Student N showed a similar trajectory. Her Grade 4 writing used varied adjectives (“暖暖”, “青青”, “各式各樣”) and compound sentence structures. By Grade 6, she employed a wider range of vocabulary (“高高”, “翠綠”, “清澈”, “小巧”) and maintained a clear spatial progression in her travelogue. However, her writing still remains at the level of visual description, with limited sensory depiction. In the final independent task, her writing, like student S, showed a moderate degree of internalisation: fluent but not rich enough. These cases indicate that the results of AI tasks are not either-or (success or failure), but are located on a continuous spectrum of internalisation levels. Students with intermediate levels of need satisfaction showed partial internalisation, suggesting that ongoing scaffolding instruction is particularly important for students whose motivation levels are neither consistently high nor consistently low.

These differences indicate that Student T had a more open attitude towards the learning experience when she entered the learning unit. She believed that the teaching aids and activities were more helpful, and she paid more attention to details beyond the teaching content. Therefore, her motivation to learn was not only influenced by the teaching conditions, but also by her initial learning attitude.

The artificial intelligence image task indicates that although the real multimodal experience is powerful, it is not sufficient to ensure the formation of intrinsic motivation. Those students who failed to receive adequate need satisfaction during the 3-year learning process—their abilities remain fragile, their autonomy has not been exercised, and their relatedness has not been deeply experienced—once deprived of assistance, they are unable to continuously create high-quality articles. For these students, the motivational boost from the early tasks is still context-dependent and has not yet translated into lasting tendencies.

This finding has important implications for understanding the limitations of technology-mediated motivation. Virtual reality and on-site visits can create powerful experiences, but these experiences must be carefully designed in order to help students internalise motivation. Without such design, the gap between top students and lower-performing students may widen rather than narrow.

## 5 Discussion

In this section, we interpret our findings from the perspective of teacher-researchers. We do not view technology (VR, Prezi, AI) or activities (field trips) as isolated factors that cause changes in motivation. Instead, we consider them as teaching decisions made by the teacher-researcher—namely, when to introduce which tools, how to build learning scaffolds, and how to respond to the constantly changing needs of students. Therefore, the following analysis will focus on elaborating the teaching concepts behind each task design, as well as the interrelationship between teachers‘ investment and students' motivation.

This longitudinal study contributes to SDT by revealing three distinct paths of motivation development (improvement in competence, enhancement of autonomy, and increase in relatedness). The formation of these paths—as well as their eventual internalisation or abandonment—is influenced by the interaction between technology and instructional scaffolds. In addition to depicting the development trajectories of competence, autonomy, and relatedness, this study also: (1) elucidates the conditional relationship between technology empowerment and need satisfaction; (2) illustrates the value of teacher-researcher design as a method for studying classroom motivation; (3) provides a practical tool for evaluating the internalisation of motivational benefits. We will elaborate on these contributions in the following sections.

### 5.1 Teacher-researcher decisions in scaffolding competence development

The evolution from “narratives” to multi-sensory storytelling aligns with the capability principle of SDT. However, our data indicate that merely having abundant sensory input is insufficient. Only when students (such as Elsha) start to actively choose and weave sensory details into a coherent narrative can the ability be internalised. The stark contrast between students in AI tasks who can demonstrate their abilities through metaphors and those who regress to general listing shows that writing ability not only includes the ability to describe but also the tendency to transform perception into written expression—this tendency requires repeated and progressive cross-sensory practise to cultivate. This finding expands Teng (2025) research on GenAI-assisted writing, indicating that the teacher-researcher must carefully arrange experiential scaffolds (virtual reality, field trips) to support the internalisation of ability.

This finding is in line with the view of Makransky and Petersen (2021), that immersive experiences alone do not guarantee learning outcomes; the integration of teaching is crucial. However, although their research focused on immediate learning outcomes, our longitudinal data further expand this insight, indicating that the effect of sensory scaffolding is not automatically produced, and without continuous teaching support, its effect may weaken over time.

### 5.2 Teacher affirmation and the negotiation of autonomy

The case of Student T elevates autonomy from the level of task selection to the level of identity negotiation. The teacher's affirmation transforms an ordinary writing task into a space for cultural construction. In a multilingual environment, supporting autonomy means recognising that learners have the right to integrate complete cultural resources into the L2 writing space. The emergence of individual voices in other students‘ writings further indicates that writing has shifted from compliance to meaning construction. This finding is in line with the research results of Zhang and Wang (2025), who discovered that in writing in English as a foreign language (EFL), feedback that supports autonomy is more likely to stimulate learners' enthusiasm than simple praise. The case of Student T shows that the affirmation of cultural capital by teachers can transform an ordinary writing task into the construction of identity—she positions herself simultaneously as a Chinese learner and a cultural inheritor.

The preceding analysis focused on the individual learner's identity work. We now turn to the mediating role of different task modalities. This extends the findings of Zhang and Wang (2025), showing that autonomous support in multilingual classrooms includes not only feedback practises, but also the affirmation of learners' cultural resources -a dimension that has received less attention in mainstream self-determination theory research.

### 5.3 Teacher-mediated orchestration of technology affordances

The task sequence illustrates context-sensitive pedagogical orchestration under changing constraints. In Grade 4, pandemic restrictions necessitated digital alternatives such as Prezi for visual scaffolding. As restrictions eased in Grade 6, VR was introduced to deepen sensory input, which was then complemented by a field trip to Kowloon Walled City Park to foster embodied social engagement unachievable in virtual environments. Rather than treating technologies as isolated interventions, the teacher-researcher selectively integrated these tools within a responsive, need-supportive ecosystem.

By the sixth grade, AI tools for converting text into images had become widespread. Considering their teaching potential and the risk of over-assistance, the AI-image task designed by this teacher-researcher was not a purely technology-driven activity, but rather an evaluative tool aimed at assessing whether students could independently apply the learning motivation developed in previous tasks. The deliberate removal of assistance measures—without teacher guidance, without peer collaboration, without prior exposure to VR or field trips—was intended to test the internalisation of competence, autonomy, and relatedness by students in a real autonomous writing environment.

Our task sequence indicates that different technologies vary in their ability to support psychological needs. Prezi provides visual scaffolding but limits sensory and social interaction. VR expands sensory input but still offers a personalised experience. Field visits, although not highly technological, uniquely facilitate shared attention and spontaneous joint construction of meaning, thereby effectively meeting the needs of interpersonal relationships. This highlights that the motivating effect of technology depends on its ability to promote social interaction. The teacher-researcher designed the subsequent AI-image tasks specifically as low-scaffold assessments to evaluate intrinsic motivation in its purest form, thereby revealing the limitations of the previous learning outcomes once sensory immersion and social scaffolding were removed.

One may question whether the observed engagement reflects a novelty effect, or an ongoing motivational development. However, our questionnaire survey data indicate otherwise. Field visits were considered very helpful (M = 4.2381, SD = 1.1360), while the evaluation of the Prezi virtual tour was more varied (M = 3.3810, SD = 1.4310). Some students even stated that it was “completely unhelpful.” If only novelty drove the level of participation, then all unfamiliar tools would likely receive consistent positive feedback. The differences among various tools indicate that students‘ responses are not due to novelty itself, but rather to the convenience provided by the teaching methods. Moreover, the final independent AI-image task shows a clear bimodal pattern: only students who consistently met their needs in previous tasks were able to maintain high-quality writing, while other students showed a decline. The pure novelty effect cannot produce such a theoretical and demand-based individual difference. Therefore, we believe that although novelty may have played a certain role in the initial participation, the continuous development of motivation depends on the extent to which students' psychological needs are supported throughout the task sequence.

For the observed patterns, there are other possible explanations worth considering. One possibility is that the differences in the difficulty of the four writing tasks might have affected students' performance rather than the development of their motivation itself. The AI-image task requires students to write without any assistance, which might be more cognitively challenging than the previous tasks that used Prezi as an aid. However, if it is only the task difficulty that led to the observed differences, we would expect that all students' performance under the AI condition would be generally lower. Some students (such as Student T) maintained high-quality writing, while the writing skills of others declined, indicating that it was the previous level of need satisfaction—rather than the task difficulty—that was the differentiating factor. Similarly, although students' Chinese language ability undoubtedly affects their writing quality, the longitudinal research design enables us to track each student's performance relative to their own baseline level, thereby controlling for stable individual differences in language ability. Therefore, the different results observed in the AI-image task more strongly point to differences in motivation internalisation rather than merely differences in language ability.

This is in line with the results of the meta-analysis conducted by (Alé and Arancibia 2025), which states that when the instructional design meets the needs of the learners, the impact of immersive technology on intrinsic motivation is more significant. Our study adds a longitudinal perspective, indicating that this conditional relationship is not only applicable to short-term interventions but also to continuous learning over a period of 3 years, and the instructional design must remain stable in order for learning motivation to be internalised.

In conclusion, these research results indicate that technology does not operate independently of teaching; digital tools themselves do not have inherent motivating effects and cannot sustain themselves. On the contrary, the motivating effect of any technology depends on the conscious teaching organisation by teachers based on specific contexts. It is the teacher's active guidance—determining when to introduce the tools, how to establish the usage framework, and when to gradually reduce support—that can transform the inherent characteristics of the technology into the psychological needs satisfaction of students.

### 5.4 Reciprocal investment: theorising the teacher-researcher contribution

We define reciprocal investment as an extension of investment theory (Norton, 2013; Darvin and Norton, 2023). It captures a dynamic two-way process: the teaching input of teacher-researchers—designing technology-assisted writing tasks, making contextualised decisions based on changing constraints, and affirming students' cultural identities—can promote students' participation and need satisfaction. Conversely, the improvement in students' motivation and engagement will stimulate the professional input and teaching creativity of teacher-researchers, thereby giving rise to more teaching innovations. Unlike the one-way theory of teacher influence, reciprocal investment emphasises the virtuous cycle of mutual empowerment between teachers and students.

This concept differs from the related construct. Teacher agency emphasises the ability of teachers to act autonomously within structural constraints, but it does not theorise the mutual influence of students‘ responses on teachers' actions. Relational education focuses on establishing emotional bonds and caring relationships, but it does not address how teaching investments generate incentive rewards, thereby reshaping teaching practise. Reflective practise, although valuable as an introspective process for examining one's own teaching, is essentially individualistic and retrospective; in contrast, reciprocal investment is interactive and cyclical, reflecting the mutual empowerment observed in this longitudinal design.

**Figure 1:**
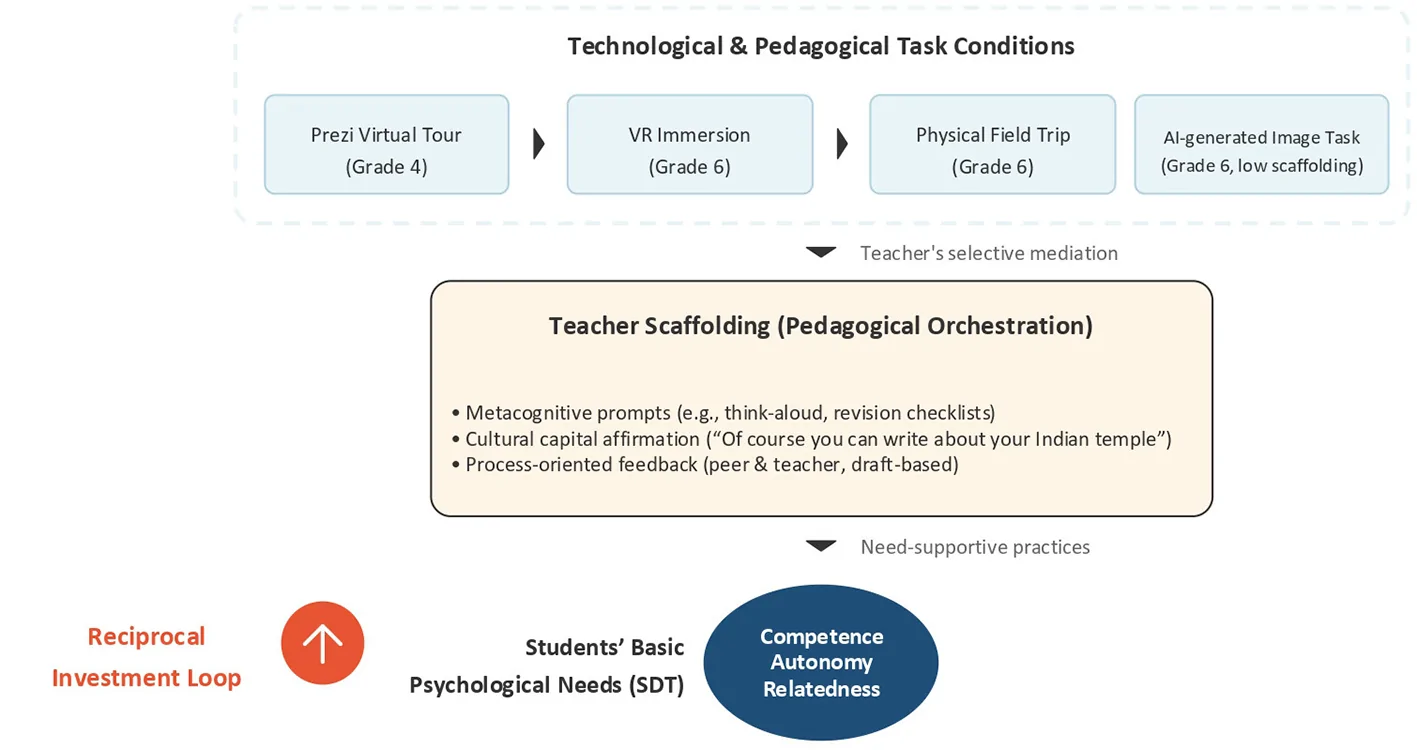
The reciprocal investment model of motivation internalisation.

The two aspects of deficiency led to the proposal of this new concept. Firstly, although the Self-Determination Theory (SDT) research focuses on how teachers support students' psychological needs, it pays less attention to how students' motivational responses affect teachers' subsequent teaching decisions over time. Secondly, the role of being both a teacher and a researcher provides a valuable perspective for understanding this two-way interaction, but this role has not yet received sufficient theoretical elaboration. Reciprocal investment addresses these gaps by providing a lens for understanding the co-evolution of teacher practise and student motivation in classroom-based research. Figure 1 summarises this dynamic process.

### 5.5 Practical implications: using AI-image tasks to understand internalisation

The AI-image tasks have a practical application: they can provide insights into which students have internalised their learning motivation and which students still require continuous assistance. For students like Student T, the artificial intelligence image tasks provide an opportunity to showcase their autonomous learning ability; while for other students, it exposes the vulnerability of their learning motivation. Teachers can not only use these tasks to assess students' writing abilities, but also identify those students whose needs for satisfaction still rely on specific contexts and may require additional support. This aligns with the suggestions for formative assessment in a technology-assisted environment (Wiliam, 2022).

### 5.6 Integrating the findings: a summary framework

The findings of this study reveal a complex interplay between basic psychological needs, the mediating role of technology and pedagogy, and the resultant internalisation of motivation. To integrate these findings, Table 5 provides a summary framework that delineates the key developmental trajectories for the three needs from Grade 4 to Grade 6, the moderating role of different technological and pedagogical conditions, and the divergent degrees of internalisation manifested in the final independent task (AI-image writing).

**Table 5:** An integrative framework of findings: need trajectories, technological moderation, and internalisation outcomes.

Basic psychological need	Key developmental trajectory (grade 4 to grade 6)	Moderating role of technology/task design	Manifestation of internalisation in the low-scaffold “AI-image task”
**Competence**	From visual, formulaic “thing language” → multisensory, affectively rich personal narrative.	**Immersive technologies (VR) and the field trip** provided crucial sensory input, enriching descriptive potential. However, **teacher guidance and scaffolding**? were pivotal in helping students actively select and weave this input into coherent, confident expression.	**Internalised**: ability to employ creative devices like metaphor (e.g., “was like a mirror”) for independent description. **Not Internalised**: Reversion to basic, enumerative “thing language.”
**Autonomy**	From completing an external task → meaning-making and identity negotiation by infusing writing with personal and cultural connexions.	**Teacher affirmation of students' cultural and personal connexions** acted as a key catalyst, transforming a standard writing task into a space for identity authorship. Technology (e.g., Prezi, VR) primarily supplied content but did not automatically elicit such deep connexions.	**Internalised**: Sustained presence of personal voice, memory, or cultural references in writing without external prompts. **Not Internalised**: Writing lacks personal connexions, remains generic, and shows limited original perspective.
**Relatedness**	From individual learning → writing that emphasises shared experience, social interaction, and the use of collective pronouns.	The **physical field trip** uniquely fostered spontaneous peer discussion, shared attention, and collaboration—social processes that directly nourished relatedness and were difficult to replicate with purely virtual tools (Prezi, VR).	**Internalised**: Ability to draw upon or recreate memories and perspectives from social experiences in the independent task. **Not Internalised**: Writing is presented solely from an individual perspective, lacking a social dimension.

Table 5 synthesises the evidence that while immersive technologies and rich experiences can effectively trigger classroom engagement and immediate need satisfaction, the **internalisation** of this engagement into a stable, transferable disposition for writing motivation depends on sustained, need-supportive pedagogical scaffolding across different task modalities. In this internalisation process, the teacher-researcher, through **reciprocal investment**—where their own professional development and classroom practise form an interactive cycle—acts as a central moderator and catalyst. This framework reinforces the core argument: motivational development is not merely about providing engaging experiences, but about purposefully designing instructional sequences that support the **internalisation of needs**.

### 5.7 Limitations and future directions

This study has some limitations. Firstly, the research subjects were limited to a single class in one school in Hong Kong, involving a total of 25 students. Therefore, the generalisability of the results is limited. Secondly, due to the lack of a control group, we were unable to clearly attribute the motivation improvement to the technology-assisted teaching tasks; developmental factors and other teaching variables may also have had an impact. Without a control group, the internal validity of the observed changes is necessarily limited, as the effects of maturation and other confounding variables cannot be excluded. Another limitation lies in the integrated design of the task sequence, which prevents us from distinguishing the specific impacts of each technology from the overall instructional design. Students experienced a combination of carefully arranged Prezi, VR, field trips, and AI tasks, each providing varying levels of support. Whether similar motivational effects could be achieved through other technology configurations—or by arranging the same technologies in a different order - remains to be investigated in future studies. Thirdly, although the perspective of the teacher-researcher provided unique insights, it also introduced potential biases. To mitigate this bias, we established inter-coder reliability (Cohen's κ = 0.8049) and regularly discussed the research results with peer researchers. Furthermore, the specific sequence of the technologies and teaching interventions examined in this paper—from highly supportive virtual tours (Prezi) to immersive virtual reality, then to collaborative physical experiences, and finally to low-support artificial intelligence tasks—constitutes the teaching model that the teacher-researcher carefully designed. Therefore, our research results on the support for different approaches to psychological needs and the evaluative value of independent tasks can be most directly applied to learning environments that adopt a design logic similar to “from support to autonomy.” Future research can compare different integration sequences or durations of technologies to find the best way to promote internalisation of motivation. Nevertheless, conducting longitudinal follow-up studies on the same group at multiple time points enables us to observe the development trajectories that cannot be captured by cross-sectional designs, although causal attributions remain limited.

From a teacher-researcher perspective, the pedagogical design principles identified here have shown promising transferability beyond the focal classroom. Subsequent reflective teaching practises by the first author in a comparable primary school setting—implementing the same task sequence of Prezi, VR, field trips, and map-making—yielded similarly positive student engagement and writing outcomes. These preliminary professional observations further reinforce the practical utility and adaptability of the proposed scaffolding model across multilingual L2 learning contexts.

Finally, although the perspective of teacher-researchers offers unparalleled depth, it also implies that the identified reciprocal investment dynamics are essentially closely related to the background of teachers simultaneously engaging in deep professional learning. Whether this dynamic can be generalised to other teaching contexts is worthy of further research. Future studies can invite multiple teacher-researchers from different schools to participate and examine how varying degrees of continuous professional engagement interact with the classroom motivation ecology.

## 6 Conclusion

This 3-year longitudinal teacher-researcher study tracked 25 NCS primary students' Chinese writing motivation across a sequence of technology-mediated tasks: Prezi virtual tours (Grade 4), VR immersion (Grade 6), physical visit to Kowloon Walled City Park (Grade 6), and independent AI-image task (Grade 6). Guided by Self-Determination Theory, the study examined how students' needs for competence, autonomy, and relatedness were supported across tasks, and whether gains were internalised for independent writing.

Findings revealed clear developmental trajectories. Competence developed through increasingly multisensory writing; autonomy emerged through personal and cultural meaning-making, powerfully illustrated by one South Asian student's connexion between Hong Kong statues and Indian temple memories; relatedness functioned as a motivational anchor, with writing reflecting collective experiences and shared emotions. However, the independent AI-image task revealed stark individual differences, suggesting that authentic multimodal experience alone is insufficient for internalised motivation without sustained scaffolding over time.

The study contributes both theoretically and practically. Theoretically, it extends SDT's application in multilingual, technology-mediated contexts and proposes the concept of “reciprocal investment” to articulate the agency of the teacher-researcher. Practically, it offers clear guidance for designing motivational writing sequences: experiential learning should scaffold independent writing; pedagogy must actively create spaces affirming student autonomy and cultural identity; collaborative tasks are crucial; and low-scaffold independent tasks can effectively evaluate levels of motivational internalisation.

As demonstrated by the case of Student T, who wove a memory of an Indian temple into her Chinese travelogue, for these NCS students, the journey entailed not only progression in writing skill but an expansion of identity and expression within Hong Kong's multilingual landscape. The enduring challenge lies in designing learning environments where such identity work becomes possible for every learner.

AI Usage Statement: During the writing of this article, the author utilised Grammarly and ChatGPT-4 for English language editing and grammar checking to enhance the readability of the text and correct grammatical errors. After using these tools, the author reviewed and edited the content as needed and is fully responsible for the content of the final draught.

## Data Availability

The raw data supporting the conclusions of this article will be made available by the authors, without undue reservation.
