# Supplementary material for: A teacher's lens on student motivation: a three-year longitudinal study of NCS students' Chinese writing motivation in Hong Kong
Source: Front Psychol. 2026 Aug 11;17:1906908. doi: 10.3389/fpsyg.2026.1906908 (PMC13503172; doi:10.3389/fpsyg.2026.1906908)
Supplement: Supplementary file 1 [file Supplementary_file_1.docx]

Supplementary_Data_Appendix

**Appendix A: Grade 4 Travelogue Unit Evaluation Questionnaire Structure**

| Item Category | Example Items | Response Format |
| --- | --- | --- |
| Overall enjoyment | "Did you enjoy learning the content of the travel journal unit?" | 5-point Likert |
| Perceived learning gains | "Do you feel that your understanding of multi-sensory descriptions improved after studying the texts?" | 5-point Likert |
| Tool effectiveness | "Did the Prezi software help you better understand the environment of Hong Kong Park?" | 5-point Likert |
| Field trip impact | "How did the field trip to Hong Kong Park help your writing?" | 5-point Likert + open-ended |
| Writing challenges | "What part of writing the travel journal was the most difficult for you?" | Multiple choice + open-ended |
| Skill improvement | "What skills do you think you improved through the field trip?" | Multiple choice + open-ended |
| Comparative insights | "What details did you discover through the field trip that were not mentioned in the Prezi VR?" | Open-ended |
| Future suggestions | "What improvements would you suggest for future travel journal courses?" | Open-ended |
| Overall rating | "Please give this unit a score." | 0-100 scale |

**Appendix B: Grade 6 End-of-Unit Culture, Identity, and Writing Perception Questionnaire Structure**

| **Item Category** | **Example Items** | **Response Format** |
| --- | --- | --- |
| **Knowledge & Understanding** | *"I understand the characteristics and significance of major Chinese cultural landmarks."* | 5-point Likert |
| **Writing & Expression Skills** | *"I can use vivid language to describe a Chinese landmark I have visited."* / *"I can write a well-structured travelogue."* | 5-point Likert |
| **Cultural Connectedness & Identity** | *"I feel a close connection with Chinese culture."* / *"Did you feel a greater sense of identity as part of Chinese culture after the VR and field trip tasks?"* | 5-point Likert / Multiple choice |
| **Modalities & Tool Helpfulness** | *"Do you think learning through VR/field trips helped you more effectively understand Chinese cultural heritage and write better essays?"* | Multiple choice / 5-point Likert |
| **Reflective Insights & Open Questions** | *"What new understanding do you have regarding your cultural identity through this unit?"* / *"What was the most profound impression left by this learning sequence?"* | Open-ended |

***Note.*** *The Grade 6 questionnaire was administered at the end of the Grade 6 sequence to evaluate students' longitudinal shifts in Chinese writing self-efficacy, tool helpfulness, and perceived cultural connectedness following the VR immersion and physical field trip tasks. Selected core dimensions from this instrument were synthesised with the Grade 4 baseline data to construct the focal case comparison in Table 4. All items were originally delivered in Chinese with teacher-researcher guidance to ensure full comprehension by non-Chinese speaking participants.*
